# Factors affecting safe emergency evacuation of subways in Iran: findings of a qualitative study

**DOI:** 10.5249/jivr.vo112i2.1259

**Published:** 2020-07

**Authors:** Fatemeh Nouri, Davoud Khorasani-Zavareh, Reza Mohammadi

**Affiliations:** ^*a*^ Department of Health in Emergencies and Disasters, School of Public Health and Safety, Shahid Beheshti University of Medical Sciences, Tehran, Iran.; ^*b*^ Workplace Health Promotion Research Center (WHPRC), Shahid Beheshti University of Medical Sciences, Tehran, Iran.; ^*c*^ Department of Neurobiology, Care Sciences and Society (NVS), H1, Division of Family Medicine and Primary Care, Alfred Nobels Allé 23 141 83 Huddinge, Sweden.

**Keywords:** Emergency evacuation, Passenger safety, Subway evacuation, Crowd disaster, Emergency crowd evacuation

## Abstract

**Background:**

Development of construction and operation of subway rail transit system in populated cities of middle- and high-income countries along with the increasing population of its users, have exacerbated the safety problems of the users against incidents and emergencies in subway stations. Although subway emergency evacuation is considered by the governments and subway network organizations as a critical task related to passengers' safety at the time of emergency, the risk of mass evacuation in station is undeniable. The main objective of this study is to identify factors affecting safe evacuation of the population from subway station and to propose the strategies for addressing them based on experiences or opinions of stakeholders in Tehran Subways, Iran.

**Methods:**

This is a qualitative study that was conducted between January 2017 and December 2018, in which a semi-structured interview was conducted for 17 participants among the senior managers, executive managers, subway station operations staff, and subway passengers in Tehran subway stations, in the Capital of Iran. In order to analyze the data of this qualitative study, the Graneheim and Lundman method was used and manifested content analysis approach was employed.

**Results:**

Based on the findings of this study, the factors affecting safe evacuation of the population from subways station were identified in four main categories covering passengers, organization, communications, and environment. Then the main categories of "culture, interaction and cooperation of passengers", "correct and timely decision-making", "notification", and "location of emergency evacuation" were emerged as safe emergency evacuation challenges and the most important findings of this study; and strategies were proposed to improve the safety of passengers at emergency evacuation of subway stations.

**Conclusions:**

In the present study, the lack of safe approach to improving passengers' safety in the development plan of Tehran subway rail transit network is a major concern for managers and operations staff. Therefore, changing the attitude of policy makers from focusing on the quantitative development of passenger transportation services to improving safety and then the quality of passengers' trip is taken into account as an urgent need to improve the safety of subway passengers.

## Introduction

Population growth as well as increasing demand for subway urban rail transit network throughout the world have changed the subway stations to one of the crowded public places. Despite its critical role in passenger traffic flow in large cities, the underground construction of subway network and the location of the stations might turn an incident to a catastrophe.^1-4^ As the 1995 Baku Subway fire in Azerbaijan with more than 286 deaths and 270 injuries, and 2003 Daegu Subway in South Korea with 189 deaths are considered to be the deadliest subway disasters in the world.^5-7^ On the other hand, the panic among the population caused by such incidents, precipitates secondary incidents increasing the destructive impact of the whole incidents.^8-10^ For instance, the sudden braking and stopping of the Guangzhou Subway in 2013 caused the passengers of the last wagon to flee to the front of the wagon with terror leading to a number of injuries due to the stampede of the population.^11^

Iran is a middle-income country, where subway has been constructed and utilized in eight metropolises including the capital city of Tehran. In recent years, developing the operation of Tehran Subway rail transit lines and its popular recognition has yielded to unprecedented levels of density and demand especially at intersections and exchange stations. Currently, Tehran Subway has owned 20% share of intra-urban transportation in this metropolis, with an average of more than two million trips per day on the five active subway lines in Tehran for more than 1,500,000 passengers.^12^ A survey of the number of passengers at some stations at rush hours in the mornings suggests that on a daily basis these stations use more than 100% of the estimated capacity at the station's design.^12^ In this situation, the actual capacity projected to evacuate the population from the station cannot respond to the actual need of passengers to evacuate in safe time; and will be problematic for the safety of the passengers.^4,12^

Regarding the probability of incidents in the subway, there is always a possibility of emergency evacuation due to terrorist activities, fire, or other accidental calamities. At the time of threat or disaster, emergency evacuation is considered as a potential measure for saving lives and an effective strategy to address the risk of flood, earthquake, explosion, and other sudden incidents.^13^ Therefore, subway safety evacuation is regarded as an important issue in the design of subway incident prevention and safety management of subway systems. This concept emphasizes the safety of population and their directing to the safe place before the damage is intensified.^14,15^

The studies on subway emergency conditions started from 1990s focusing on evacuation capability assessment in an emergency, emergency evacuation strategies, emergency situation, and establishment of an emergency system.^11,16^ The focus of the previous studies has mostly been on identifying the passengers in need, evacuation routes and safe zone areas at the stations, and safe time to evacuate the passengers,^4,17^ or a few publications have investigated improving the management processes at the strategic level with the priority of safety and service quality.^18-20^ Previous studies have indicated that stakeholders experience can improve the phenomena of interest for better management. However, the lack of qualitative studies is still evident in terms of discovering the stakeholders' experience and affecting factors on the safe evacuation of the population from the perspective of managers and operations staff at the stations. Therefore, the present study tried to identify factors affecting safe evacuation of the population from subway station and to propose the strategies for addressing them based on experience or opinions of stakeholders in Tehran Subways in the hope of filling this study gap. 

## Methods 

This is a qualitative study design, in which a content analysis approach was employed. Content analysis was used to analyze data and interpret the meaning in order to explore the affecting factors, barriers, facilities, and description of the studied phenomenon.^21^ As the philosophy of qualitative study, since little known about emergency evacuation in subways, it was decided to use the qualitative study. Among various data collection method, both face-to-face interview and observational field notes was performed. Face-to-face interview is suitable when researcher want to saturated concepts emerged from other participants, observation or Focus Group Discussion (FGDs). Moreover, since this study was conducted for the first time in Iran, there was a lack of knowledge on effecting factors on safe emergency evacuation. And authors of this study were looking for individual experiences in the context, subways in Tehran, Iran. The interview allowed authors to extract concepts from the participants' experiences and could explore a lived experiences of stakeholders in the emergency evacuation of Tehran subways and interview was the suitable method to saturate concepts based on participants' point of view.

**Setting**

Tehran is the capital of Iran and based on latest census data of 2017, it has a population of about twelve million people. Every day over 5 million population in Tehran used public transportation including bus rapid transportation (BRT), taxes, and subways. The Tehran subway has an important part of public transportation by five active lines.

This study was conducted based on the experience of stakeholders in emergency evacuation of subway stations in Tehran metropolis. In this regard, participants with theoretical knowledge or practical experience at Tehran Subway stations, administration offices, Tehran Urban and Suburban Railway Company (TUSRC), Crisis Prevention & Management Organization of Tehran Urban Railway, as well as subway users were interviewed for data collection.

**Participant selection and data generation**

In this study, firstly the identification of key informants was performed by FN who has been researching in the field of Health in Emergencies and Disasters since 2013 and DKZ who has about 16 years of experience in the same field as well as safety promotion and injury prevention. Moreover after fourth interview theoretical sampling technique was used to identifying key persons who has well experienced in subway emergency evacuations based on key informant introductions. Therefore, 13 officials from TUSRC with practical experience in evacuation of subway stations at administrative and operational levels were interviewed. On passenger's selection, verbal people who regularly used subway for travel in city and were interested to participate or well communicated selected. Finally, 17 participants were enrolled in the study which consisted of 4 senior managers, 6 middle and operations managers, 3 operations staff, and 4 passengers who used the subway. The number of participants were based on the saturation principle.^22,23^ In overall, 65% of participants had an age range of 35-45 years, and 54% had a work experience of over 11 to 20 years (Table 1).

**Table 1:** Demographic characteristics of participants in the study of factors affecting the safe evacuation of the population from subway stations, Tehran, Iran.

Participant characteristics		Number	Percentage
**Gender**	Male	13	76
****	Female	4	24
**Age (Years)**	35-45	11	65
****	46-55	4	23
****	>55	2	12
**Interview level**	Senior managers	4	23.5
****	Middle managers	6	35
****	Operations staff	3	18
****	Passengers	4	23.5
**Education**	Bachelor's degree	10	59
****	Master's degree	6	35
****	Ph.D.	1	6
**Work experience (Years)**	<10	1	7.7
****	11-20	7	53.9
****	>20	5	38.4

Initially, two unstructured interviews were conducted to get overall concepts. Then, 15 semi-structured interview in Persian was used with interview guide for data collection and during the interview, each new concept was saturated. Face-to-face interview was conducted for each participant individually. The managers and staffs were interviewed at their office and passengers were talked at any places of station that they were comfortable and interested. Interviews started with general questions on the national approaches for subway station evacuation and continued with more specific questions on the national strategies for barriers of emergency evacuation of subway stations, and suggestions to improve population safety at the time of evacuation in disasters.

The main structure of the interview consisted of the following questions: What do you think of the factors that make safe evacuation effective? Based on your experience, what challenges did you encounter in the evacuation of the station? What do you think of the barriers of the station evacuation? Based on your experience, what would you recommend to improve the safety of station evacuation? Then the concepts came up with questions like how, why, please explain more to reach saturation level.

All interviews were recorded through a digital recorder and then were transcribed verbatim by FN as a principal investigator. Only in one case, upon request of the participant, the voice was not recorded and the full interview was written by the principle investigator and then transcribed verbatim and finally approved by the interviewee. The interviews lasted for about eight months from 20June2017 to 23January2018, and the interview time was 25 to 60 minutes with an average of 48 minutes, and durations of more than 90 minutes in two cases.

**Data analysis**

The Graneheim and Lundman method was used to perform the manifested content analysis.^24^ Accordingly, following five steps including: meaning unit, condensed meaning unit, code, subcategories, and categories were performed.^24^ All recorded interviews were immediately transcribed verbatim into Persian at the first day after the interview and then typed by Microsoft Office 2007 software. To fully understand the content of the interview, the researcher FN first listened to the recorded file for several times and fully transcribed it, and then the full text of the interview was repeatedly read and the meaning units and initial codes were extracted. By DKZ supervision, the similar initial codes were classified in broader categories and finally the main categories were determined.^24,25^ In this study, data collection and data analysis were carried out simultaneously and the participants' point of view about the less-addressed categories was questioned through repeated complementary interviews or new interviews with next individuals. In order to obtain an overall understanding of the interview, the first author repeatedly listened to the interviews, and after transcription and typing, read the final text frequently and checked with the recorded file to form the initial codes. Then, in coding stage, all interviews were repeatedly read and key words and phrases associated with the research question were extracted. Then, based on the similarities and differences of the codes, they were compared by the FN and DKZ and then the categories and subcategories were formed. For the first six interviews, the main researcher examined the interview questions and summaries of the interviews with the research team and, based on the vague points and gaps in the subcategories, additional questions of the subsequent interviews were selected. The process of collecting data continued until concept saturation.^22^

**Ethical consideration**

This research, as a part of a Ph.D. thesis, was approved by the Medical Ethics Committee of Shahid Beheshti University of Medical Sciences with the code of IR.SBMU.RETECH.REC.1396.201.^26^ Moreover, all necessary and official permissions were received from the General Directorate of Security and Human Resource Protection of Tehran Urban & Suburban Railway Company.^12^ The subjects voluntarily participated in this study, being aware that their information would be confidentially and anonymously used in the study. At each interview, this information and the purpose of the study were provided verbally to the participants and the written consent was signed by them.

**Rigor**

In this study, trustworthiness was achieved through credibility, transferability, dependability, and confirmability. The analysis of the findings, with honesty and trust in the data, was attempted to provide an accurate picture of the factors affecting safe evacuation of the subway, by means of credibility. In order to obtain credibility, member check and peer check were done in data collection and analysis, and the barriers and restrictions of data collection were mentioned in methods section. Prolong engagement in collect and data analyzing were another way of reaching credibility. After analyzing the findings, two participants were contacted and a summary of the initial results was sent to determine whether these results were in accordance with their experience, by means of peer check. Parts of some interviews, the initial set of codes, and categories were qualitatively investigated in research team by the first author and colleagues, expert check. Constant comparison analysis was performed during the analysis to ensure and confirm the categories by the researchers. 

To achieve transferability, it was attempted to describe the study methods in details so that the readers can search for similar evidence in their own setting. In this research, the purpose of dependability was not to repeat the findings in a similar subject or context; rather attempts have been made to consider different perspectives at the research team and data analysis process through investigator triangulation.

The researchers tried to take a neuter approach to the data, and expressed their views on the findings through memo, as well as bracketing to reach confirmability. Selection and contact with the qualified participants were carried out by first author with experience in the field of health in disasters and emergencies and injury prevention. Subsequently, the participants with practical experience in evacuating the subway station were introduced to the researcher by their colleagues or the station manager. The interviews were conducted in Persian language and were transcribed, coded, and analyzed in Persian, then translated into English. The three researchers were fluent in Persian and English.

## Results

As shown in Table 2, the effective factors in safe emergency evacuation of the population at the subway were identified in four main categories including passengers (with four subcategories of culture, interaction and cooperation of passengers, passengers with special needs, and congestion level of pedestrian flow); organization (with five subcategories of correct and timely decision-making, incident command, organizational preparedness, fatalism, and resources); communications (with two subcategories of notification and coordination); and environment (with three subcategories of infrastructure, potential risk of evacuation, and location of emergency evacuation).

**Table 2:** Factors affecting safe emergency evacuation of the Subway based on stakeholders experiences in Tehran, Iran.

Theme	Subtheme	Examples from the code		
**Passengers**	Culture	Folk culture challenge	Lack of subway etiquette	Lack of respect to the others' rights
	Interaction and cooperation of passengers	The necessity of passengers cooperation	Lack of passengers cooperation	he necessity of cooperation with the terrified people
	passengers with special need	Lack of attention to the elderly and people with impairments	Helping disabled, blind, impaired, and elderly people exit	The risk of limited flow of disabled and elderly people
	Congestion level of pedestrian flow	Blocked access by passenger congestion	The challenge of passen-ger congestion at the stations	The challenge of decreasing passenger load at the stations
**Organization**	Correct and timely decision-making	Lack of holistic decision-making	Lack of timely decision-making	Arbitrary decision-making
	Incident command	Not having scientific crisis management	Lack of familiarity with the function of ICS	Lack of obedience to the operation commander
	Organizational Preparedness	Lack of emergency evacuation plan	Necessity of station preparedness for emergency situations	Failure to assess station pre-paredness level
	Fatalism	Fatalistic attitude of the man-agers	Considering the incident to be far from us	Fatalism in emergencies
	Resources	Necessity of having trained personnel	Serious defect of tele-communications equipment	High cost of renovating navigation equipment
**Communication**	Notification	Necessity of timely notification	The challenge of infor-mation accuracy	Way of notifying passengers
	Coordination	Necessity of coordination with relief organizations	Signing agreements with Bus Company	Necessity of coordination of the station personnel
**Environment**	Infrastructure	Quick access to the street surface	Not having emergency exit	Not having secure gathering place in some stations
	Potential risk of evacuation	Risk of crowd congestion	The challenge of height difference of train from the floor at tunnel evacuation	The challenge of power outage and having no light
	Location of emergency evacuation	Importance of evacuation time	Necessity of identifying safe places at the stations	Importance of passenger evacuation at the nearest safe place

**Passengers**

**Culture**

A majority of middle and operations managers of TUSRC believed that subway industry in Iran has emerged without preparedness and training the etiquette of using it. These participants considered the lack of a prevention culture and lack of promoting passenger transport etiquette as weak points of culture management, which in the subway industry has led to neglect of developing the etiquette of using subway. Considering the participants' experience, lack of subway etiquette is one of the major challenges of population safety during both normal and emergency evacuations. According to the participants, the etiquette of using subway includes proper etiquette of riding the subway, the way passengers interact with each other, as well as the interaction of passengers with the station personnel.

P12 "Subway was a new industry brought to Iran (by the authorities), without the etiquette, we always first bring the tool and then its etiquette of using ... Unfortunately, the authorities does nothing, for example, to step up to show the subway to the people so that we can promote our subway etiquette through such culture making.”P09 "... Observing safety is related to first, public culture and second, management culture of the society."

From the viewpoint of most subway riders, lack of respect for others' rights, aggressive behavior for entrainment and detrainment, hurrying to enter or exit, and neglecting vulnerable people and those with impairments, have become inappropriate behaviors for subway passengers that can disrupt normal evacuation, especially at emergencies. Hence, habitualizing the etiquette of walking from the left side of the stairs, standing on the right side of the escalator, and leaving the way open for more hurrying passengers, leaving the way open for the passengers to detrain, and prioritizing access and egress of passengers are the challenges of proper etiquette of riding the subway.

P12 "There's nothing as courtesy in the subway. One is carrying a baby, one might be blind (who do not receive care or attention) ..." P12 "When we ask the passengers not to block the doors and let arriving passengers alight, they do not care ... One falls down the gap between the subway and the platform and we pass over him..." P01"We Iranians have a sense of hurrying ... it must be taught, practiced and habitualized to let the passengers flow from the right side and the left side ..."P05 "Those who are healthy try to get out of that situation as soon as possible."

According to the experience of most participants, folk culture, the culture practiced by people with a low social level and literacy, the culture of prying, the need for direct observation to believe an event, and the interference of people on the scene are the most important challenges regarding weak points of passengers' culture in terms of interaction at the time of evacuation. Also from the viewpoint of some participants, social culture of the region where the subway station is located affects the level of passengers' interaction with the station's agents at the time of emergency evacuation.

P08 "Some lines have specific users that come from the other cities to do their official jobs and return to their city; the level of education is very influential, and we are more afraid that those subway users would be there at the time of emergency evacuation. What would happen to those people!? A man uses the subway with his disoriented wife and children and wants to help in emergency evacuation. More than trying to save his soul, he is struggling not to lose his family. Now I would call it the folk culture."P11"... Passengers do not trust in subway colleagues, won't believe (an incident) unless they see with their own eyes." P08"... We tell the passengers not to stop here, go that side; (instead of collaboration) they take photos, videos, and ask why the route is closed."

**Interaction and cooperation of the passengers**

According to the participants in the study, the following were listed as the challenges to evacuate passengers from the danger zone and direct them to the safe place: lack of attention to the warnings and notices announced from the station loudspeakers, failure to comply with the commands of the station's officers, as well as lack of trust and lack of cooperation of the passengers with the station's officers or relief forces. Therefore, effective interaction of the personnel with the passengers such as appropriate tune of speaking with the passengers in controlling their stress, reducing concerns, maintaining calm, and managing their excitement could influence safe evacuation of the population.

Moreover, one of the serious challenges of subway authorities in establishing effective interaction is unpredictable behavior of the passengers in accidental conditions and their lack of cooperation. Based on participant's perception, people's subjective assumptions affect determining the threshold of tolerance, reaction, and behavior of the population against unexpected events, and their cooperation in emergencies.

P06 "... On the subway, we deal with human factors; human factors are unpredictable things." P07 "... Passengers excitement must be managed, a proper paging can control a lot of passenger excitement."P11 "... We were going to evacuate the station, whatever we did, we could not lead the people, they did not come, they said: ‘we would stay here until the train arrives even if it lasts until midnight'. There was no train and the line was closed but they did not believe."P12 "When our line is out of order or we have problems, (people) instead of cooperating, worsen (their behavior); they pull emergency stop handle; open the door to get off the moving train, causing train delays and safety failure."

**Passengers with special needs**

Everyday subway stations are the place of the flow of a massive crowd of passengers with special characteristics. From the perspective of most participants, the specific characteristics and needs of passengers are of affecting factors on the vulnerability of the evacuee at the time of normal and emergency evacuations. People with special needs are meant to be those with visual, hearing, and motor impairments, the elderly, pregnant women, and children. The limitation of motion and special needs of this group of passengers challenge quick and safe evacuation of the population in normal situation and particularly emergencies.

P05 "Our passengers gather in a train from a wide range of social backgrounds; infants, children, the elderly, disabled people and those with physical and motor impairments. At the time of evacuation, the ordinary people who are physically healthy exit quickly. While people with impaired abilities get under pressure and injured ..."P12"... Among the passengers there may be a blind person, someone should help, (in an emergency) we should think for the disabled and blind people."P07"We have three types of passengers, we have no problem with healthy people, we must help the elderly, impaired, disabled, and blind people to exit the station ..."P01"... If an elderly person falls, there is the risk that others (at emergency evacuation) will collide with him and fall as well".

Most participants, in addition to age, gender, and motor characteristics of the passengers, considered lack of professionalism of passengers for emergency conditions as one of the characteristics of the evacuees, which largely affects quick and safe evacuation in emergencies. The feature of professionalism is applied to the passengers who routinely commute between certain stations and are fully aware of how to commute between the subway lines and how to reach the nearest exit at the stations. Therefore, having professional passengers for emergencies is one of the effective factors emphasized by the participants for the safe evacuation of the population in emergency situations.

P13 "... That's right, most of our passengers are professional, but for ordinary conditions only when they know where to get off, where is closer to the exit to detrain, where to stand so that the door opens in front of them, the time of trains arrival, and so on. However, in a critical status, none are professional."P08 "... A passenger who once has come to Tehran (using the subway to reach his destination) to do his official jobs and return, is not a professional one; you tell him to go out, he does not know what exit to go; he loses his way." 

**Congestion level of pedestrian flow**

Based on the experience of the participants, the gathering of passengers waiting for the trains on the platform and blocking the exit for in-train passengers have always been a challenge of the ordinary evacuation of passengers. This crowd congestion seriously challenges emergency evacuation by blocking the means of access and egress. Consequently, managing the passenger load of the stations, traffic engineering of pedestrians at the stations, and, in general, congestion level of pedestrian flow, and minimizing the crash of the moving crowd, which are effective factors of safe evacuation.

P11 "... In passenger evacuation in terms of population traffic and safety, crowd gathering itself is regarded as non-safety."P12"... The crowd has been waiting for the train for twenty minutes and it is overcrowded, the front of doors are blocked due to crowd congestion." P01 "... When the time interval between the trains is extended, the crowd is constantly growing leading to congestion and long queues ... Reducing the crowd load will facilitate passengers' movement and evacuation."P06"... When the crowd load of the station is lowered, the safety of evacuation rises making it easier to evacuate the passengers."

Based on participants experiences, high traffic volume at intersection stations, reduction of traffic capacity of the corridors and exits, the traffic of commuters between subway lines, dense outgoing population, as well as traffic congestion on the street are the factors, which result in disturbances in pedestrian traffic at the station making a serious threat to evacuating the population in emergencies. Therefore, reducing the time interval between the arrival of trains is recommended to prevent crowd congestion on the platform and have light flow of passengers at the station.

P09 "(For instance, the Station of) Imam Khomeini is one of our busiest stations; it is an intersection station, and at a moment thousands of people are passing either on the ground or through the subway. Once, the time interval between the trains at this station was 15 minutes, now it is 4 minutes. We should still lower this time based on passenger flow to have light population traffic and avoid congestion which results in rapid transit of passengers through the lines. Now, if an unexpected incident occurs, and this flood of population decide to come out, will this crowd come out healthily? I don't think so.”

**Organization**

**Making correct and timely decisions**

Regarding the experience of operations managers in emergency evacuations, correct and timely decision-making has yielded keeping golden time of evacuation and performing it with the least possible damage. Accurate decision is defined as making scientific and operational decisions with a holistic approach to solve the problem. Following this, integrated decision-making, and action based on the decisions of the station crisis manager seem to be essential to perform safe evacuation in emergencies.

P05 "... In the incident of ingress of water to the station, it was timely decision of the station's officer that helped not missing the golden time of evacuation and safely evacuate the passengers from the station and the tunnel." P10 "... A challenge, in my opinion, is the decision-making issue, many decisions are not made on time." P12 "Now everything (decisions) has become arbitrary. We never set a meeting to gather and discuss the issues, what are your comments, the station affairs?! Is this operational in your opinion?! Compile the results, set some regulations. Unfortunately, such things (consensus in decision-making) are not well performed. Someone comes and orders for something and allows, manager is changed and the (new manager) will not allow."

Most of operations managers of the stations believe that the following can be listed as the main challenges of decision-making: lack of decision-making by the senior managers due to the fear of being criticized, the necessity of decision-making for appropriate action in emergencies, the necessity of decision-making for using emergency exit and evacuation, arbitrary decisions, lack of consensus among senior managers, and lack of holistic decision-making.

P08 "... One thing that is a general problem for all the country is that, in my opinion, we are often scared to do something or make a decision that ends up as a mistake. Well it might be (a mistake). But we do not decide for the fear of not working decisions. Tomorrow if they ask we would say I preferred not to say anything."

**Incident Command**

In incidents and emergencies, unity of command is one of the effective factors in reducing harmful damage and safe evacuation. According to the experience of the participants, the arbitrary actions of the units and personnel before the command of the operations commander and failure to comply with him mean that there is no crisis management in its true sense in emergencies. Therefore, most participants considered Incident Command System (ICS) to be essential for subway stations.

P08 "We do not have something as crisis management on the subway. If a crisis occurs, we cannot manage the consequences of the crisis. The instructions tell us that in case of an earthquake all must inform the command center, and a series of measures must be done, but in practice, when the earthquake came, all escaped."

Most participants considered the efficiency and performance of the safety and crisis management units on the subway to be all show. Despite the development of an emergency operational plan for the stations (by Crisis Prevention and Management Unit of Tehran Subway), in practice, not obeying the commander, lack of an emergency squad team, and lack of emergency decision management were the major challenges of subway station officials in an emergency. Therefore, according to their own experience, an amendment to the incident command structure, familiarity with risk management and decision management for the subway station officials are essential in order to improve crisis management in emergencies.

P10 "Another subject is familiarity with risk management for the personnel in authority, for the managers … we were late in risk management as well, all of these go back to the safe management concept, these are all (risk management, decision management) the subcategories of safe management. We do not have risk management at all or maybe it has been least noted."P07 "One who comes to these positions must know risk management and know what to do in case of an incident and how to manage the risk that is imposed, or even be able to recognize the risk..."

**Organizational preparedness**

Uncertainty of operations managers about station preparedness to encounter emergency evacuation and lack of emergency evacuation plan are the preparedness challenges of TUSRC in unexpected events and emergency situations. Most authorities of the busy and high-traffic stations (such as intersections and exchanges) mentioned having no plan for evacuation in normal situations as the serious challenge of their station. Based on participants, development of an emergency evacuation plan for subway stations and passive defense of the stations seem to be essential to prevent the spread of damage and the crisis of emergency evacuation. It is also important to have a road map and a perspective of safety in long-term planning to predict bottleneck capacity development, identify alternatives for emergency evacuation, and predict emergency evacuation strategies at each station.

P15 "We have no plan for normal evacuation, and none for emergency evacuation ..."P08 "We had no plan (for safety promotion), repeatedly changing the managers has made the managers plan for two or three years of their management (they have no long-term plan). Managers do not even think about the long-term plan, saying: I will be here for two or three years, no need to think and spend for the next five years."

In addition to developing an emergency evacuation plan, writing/documenting the plan and practicing it in simulated incidents and assessment of the actual level of station preparedness are considered as effective factors to improve the efficiency of the emergency evacuation plan and execute it in the outbreak of a crisis to minimize damage. Currently, development of the emergency operational plan for the subway stations of Tehran, practicing evacuation preparedness at special ceremonies, and using emergency exits for evacuation at special ceremonies are performed as emergency evacuation practice and preparedness.

P09 "To practice any skill, you need to perform some maneuvers to get prepared for real situations (crisis). For this issue, some maneuvers must be performed, the situation must be practiced. At each station, real preparedness should be assessed. It shouldn't be like when it happens, half of the population will trample underfoot and half be killed; these are very important."P04"Emergency exit has been only used in our special events (the day of the Islamic Revolution victory, martyrdom of prominent social people, death of politicians and artists.), in which thanks God nothing unpleasant happened. In special events we practice the ways of calm evacuation of the population, and we predict the situations so that if the relief force wants to enter our system, it will be easily done".

**Fatalism**

Some participants consider fatalistic attitude of subway senior managers and considering the incident as to be way far and will never happen to us as the reasons for neglecting safety actions for subway stations and also a barrier to promotion of the quality of passenger transportation. The dominant policy of TUSRC has been maximum passenger transit, while passenger safety improvement has been less taken into account. In the period of their administration, most managers focus on establishment of new lines and quantitative development of subway lines, while the safety of passengers and the quality of trips have often been ignored.

P12 "We trust Allah, only Allah helps can us to carry the passengers, in case of a crisis we are all unable to manage it." P07 "They utilize a line without ATP system; open the station without an air handler, because they believe that nothing happens at present, we open the station. But if something happens and we are not able to manage it, there will be a lot of casualties."P08"(The managers) claim that we managed to set up 100 kilometers of subway during the period of our management, but at what cost? To what extent of risk acceptance for the passengers? The prevailing priority is initial setup of the projects, the rest of the sections will be further completed. But in practice this does not happen and the total country is run based on trust in God attitude."

**Resources**

Physical, human, and financial resources of subway stations are among the most important factors in the safe evacuation of the population in emergencies. Based on the participants point of view, according to the safety principles, it is essential to equip the station with physical resources such as firefighting equipment, emergency evacuation equipment at the train and station, personal safety equipment, fixing the defects and reinforcing public page system, appropriate ventilation system including sufficient ventilators, equipment of emergency lighting, and emergency lighting system reinforcement. Also, installation of guide signs and emergency exit guide in standard and legible sizes, exit marking indicators along with the exit sign for the blind are among the essential resources to facilitate and accelerate emergency evacuation. Considering the experience of the participants, the defective equipment of the relief forces with unsuitable function for in-depth ground is a serious challenge to physical resources.

P09 "One of our challenges which is a danger in case of emergency evacuation, is the emergency lighting system; that is, when, for whatever reason and at any time, the station's power goes off and the crowd wants to go out, the emergency lighting should illuminate the emergency exit route so that the crowd can exit with the least damage. At present this system is faulty in some stations. "

Recruiting specialized and trained personnel who are aware of their own duties in emergencies seem to be essential to remove the defects, establish safe conditions, and direct the population to safe or out-of-station places. According to the participants, despite the smartness of the ventilation system at the stations, in many cases, familiarity of the personnel with the design, facilities, and infrastructure of the station as well as the skills of the technicians in working with equipment and facilities have played a vital role in smoke control and prevention of damage to the population. Also, most of the participants believed that training rapid response teams from the stations personnel for relief aid, assisting, and directing the crowd at the time of the incident are of high importance. Finally, in terms of financial resources, most of subway senior managers pointed out that lack of financial resources has always been one of the obstacles to providing equipment and full implementation of safety plans at the stations.

P07 "If the logic of the ventilation system of the station does not work, the person (station technician) should be skilled enough to know which air handler to exhaust and which to intake to control the smoke and send it away from the passenger environment. However, this ventilation system has not been set up, if smoke occurs, it must be evacuated from the same entrance to the station. One of the leading issues is the mastery of the personnel of the company in the operation of the ventilation system for smoke control....P07 The issue was that there would be a common radio network for the entire city of Tehran. The TETRA Trunk Network system, the fire department, the municipality, the subway would be all TETRA, so that these systems could be connected, but it was too expensive and did not run in time."

**Communications**

**Notification**

Based on the experience of the participants in subway evacuation, the challenge of communication and information is one of the affecting factors in the management of incidents and emergencies. Announcement of the incident or a technical defect in an accurate, prompt, and timely manner could be effective in prompt initiation of operational, relief, and support actions and removal of defects in the system, as well as improving the performance and cooperation of subway passengers. In many cases of technical defects in trains or the impossibility of serving passengers and the need for the station evacuation, failure to correctly and promptly inform the various levels, including informing the command center, driver, relevant units inside and out of the organization, and the passengers leads to worsening of emergencies or changing an incident to a crisis. Operations personnel mentioned that when none or poor notification and attention, or the lack of standardization of general notification conversations accompanies the lack of awareness of passengers about their role in emergencies and subway environmental threats, it might lead to safety breach, lack of understanding of the risk, and increased financial damage and casualties during emergencies.

P08 "... We are afraid that the train paging system is broken and the passengers are not informed about it. A panic attack occurs. People open the door. Passengers entrance to the railroad is a threat for their life … We need to inform the passengers that dear passengers we are trying to fix the problem; knowing this will make them more patient ... Sometimes there is an inappropriate notification, We have a term called conversation standardization; Last week, the station page system announced that: ‘Dear passenger, soon the train will be boarding at the opposite platform.' This statement made the passenger say at that platform?! Well, there is no train as I can see, so I will cross the railroad to get there. Sometimes the meaning of what is paged differs for the listeners. The semantic load of the words we use for the passengers and the use of short and precise sentences matter a lot".

Permanent defect of the telecommunications systems of the trains, disconnection of radio communications, and poor mobile network coverage at several points of the tunnel are among the factors which, in case of an emergency, will lead to defective communications with the command center, the driver, on-the-shift drivers, the operations and relief teams, and the passengers. According to the participants, the following items can be listed as the serious challenges of communications in emergencies: no common communication network (common radio frequency) between subway organization and urban relief organizations, disconnection of the radio communication, and the impossibility of radio calling of subway forces and relief forces.

P10 "... When we get into trouble and ask the driver to inform the passengers that this is just a short stop and a defect that soon will be fixed, so that they wouldn't become worried, most of the trains have problems in telecommunication, radio, and public address (the means of communication between the train driver and the passengers). ... We always have a problem with the communication."P07"... The radio system of the subway and the urban operations and relief teams are not shared ... They cannot see each other on their radio systems; for example, our radio frequency network is UHF, fire department's is VHF, so they cannot see each other. Their radio units does not work at the subway station. The operations teams on the subway are not linked together ..."

Information accuracy is one of the important challenges of communications. According to the participants, receiving information from a reliable source and information accuracy are of main prerequisites for proper action in emergencies. The participants believe that establishment of a hotline with the fire department, emergency, and relief agencies is an effective measure to be taken by Tehran Subway to exchange reliable information and expedite the notification of incidents and emergency situations to the relief organizations.

P07"... We established a hotline with fire station to make them sure about the accuracy of the information and that the subway command center is reporting an incident. There is no false call, when the subway hotline calls, it's the subway command center announcing an incident and its severity. There is smoke, an emergency evacuation, the number of passengers in the train, they (the city's relief team) bases this information on and acts ..."

**Coordination**

Most of the participants argued that intra-organizational coordination is one of the challenges faced by the managers of the subway station for emergency evacuation. The coordination of the command center with the operations units, the driver, and the station manager is an important factor in timely and appropriate evacuation of the population. According to the experience of operations managers, the lack of coordination between the personnel of the station units (including safety, security, platform agents, and other coordinating personnel), as well as operations individuals on the scene delays and challenges crisis management and emergency situation management.

P13 "... It is important that what coordination should be made between the staff. When I interfere in your job it makes chaos; the challenge of poor coordination is too much. This occurs in lower ranked individuals; those who are on the scene."P03"Coordination is very important. Coordination of the station manager with the command center, with the train driver, and with the station technician ..."

Based on the participants, signing agreement and joint practices with relief and service organizations outside the subway such as emergency services, fire department, telecommunications, the bus company, urban and suburban railway company, police forces, and broadcasting organization is considered as inter-organizational coordination. Regarding the experience of most participants, lack of an organized public transportation system prepared for emergencies outside subway stations for transportation of the evacuee, is a cause of gathering and disruptions in safe evacuation of the population.

P11 "One of the areas to be coordinated for the safety is that there must be means of transportation like taxi, bus, and minibus for the passengers who are directed out of the station to avoid gathering, concern, and wandering ..."P04"Regarding emergency exit, other organizations should help as well as the bus company. When a passenger is directed to the street expects a car or bus; for emergency situations Public transportation should be prepared to avoid crowd congestion ..."

**Environment**

**Infrastructure**

A majority of the participants believed that in recent years the development of subway lines and the operation of the stations have been conducted with the least concern about the provision of infrastructure regarding population growth and passengers' safety. A number of shortcomings associated with Tehran Subway infrastructure are as follows: failure to implement infrastructure based on the design such as ventilators or exchange stations, failure to execute emergency exit for the stations and tunnels, failure to implement Automatic Train Protection (ATP) system in some lines, lack of installation of an air handler box in some stations, long distance between the stations, and the complexity of emergency evacuation. The mentioned challenges threaten the health and safety of the population under normal and subsequently emergency conditions.

P07 "For example, the ATP system is one of the priorities, which is so vital and essential to us as our guidelines say that the train motion is prohibited without ATP, but ATP system was not prepared. The circuits, signaling, mimic panel of command center are not accessible, there is no view, and no permission to work at present, but the line is in use... Unfortunately, in some places, the ventilation system has been removed and even the air handler box is not owned to run the air handler inside... When it is not executed in one station, consequently the north and south ventilators are not running. Its north and south ventilators are shared with the previous and next stations, so if a station in this middle is skipped, the next stations will lose two of the ventilators between them and one of their next station's ventilator".

Due to the inappropriateness of the capacity of the passages and corridors with population growth, the current infrastructure at most stations, especially intersections, is prone to a stampede and crowd disaster during emergency evacuation. Thus, considering the specific geographical conditions of the city and the regional population in which the station operates, the development of infrastructure, determining new alternative capacities for passengers' evacuation such as a second entrance and having at least two entrances and two exits are required as a minimum for safety promotion of emergency evacuation.

According to the experience of the participants, the absence of separate exit routes, the absence of an independent emergency exit for each line at intersections and exchange stations, or even the failure to implement the second exit at most stations have been recognized as the main challenges of emergency evacuation of subway in Tehran. Identifying safe exits, safe places at the station, emergency escape routes, and emergency exits in the infrastructure of each station could be vital measures for safe evacuation of the passengers. All participants mentioned the lack of implementation of emergency exits in some old lines of Tehran Subway, as well as non-implementation and non-utilization of the emergency exit (owing to lack of land acquisition) simultaneously with the construction, opening, and operation of the station as the serious challenges of emergency evacuation of stations.

P05 "The main part of the Line 7 is inside the highway. Due to the special geographical conditions of that area, only in the western part some stations have entrance and exit. In its eastern part, because of lack of land acquisition and the limitations for the structure of construction in the area (the buildings) its second entry has not been implemented yet ... Some emergency exits of the tunnel has been carried out, but we have stopped it at its surface acquisition and at present most of the emergency exits do not have a way to the street level."

**Potential risk of evacuation**

Subway stations, which are generally built deep into the ground, are faced with high population traffic every day. From the perspective of participants, there is a potential permanent risk at population evacuation that affects the safe evacuation. Considering the fact that a majority of station installations and facilities of exit such as escalators and elevators stop in case of power outage; the power outage and darkness in the deep earth is a potential risk of evacuating the population in an emergency. Other potential risks of evacuation were listed as the difference in the height of the train wagons with ground level n in tunnels, space limitations of the stations in the deep earth, difficulties of access to open space, and difficulties of smoke exhaust and ventilation. Therefore, it is important to identify the evacuation risks of the station and analyze the prerequisites for the occurrence of an incident in the subway in order to have safe evacuation of passengers.

P01 "... The movement of this crowd who are daily transferred between the subway lines is inherently risky. There is always the risk that at the time of arrival or departure, this massive crowd might cause mass gatherings incidents, even though there is no emergency evacuation."P07"The train on the platform is level at the platform, but in the train tunnel there is a height difference of about 120 to 130 cm above ground that makes it more difficult to get emergency evacuation; this is still for normal situations without smoke. In low-light conditions, the darkness of the tunnel and the smoke make evacuation of passengers much more complicated ... In ground stations, because there is no difference in height, there is fresh air, and it is easier to get access to the relief forces, hence less risk for evacuation."

**Emergency evacuation**

According to the participants, effective factors in expediting passenger departure and reducing the risk of evacuation in an emergency were listed as time of evacuation including peak times and special occasions; place of evacuation on the platform or inside the tunnel; type of station: intersection, exchange, normal; and the specifications of the station. Considering topography of the area and the geographical location of the station, its design and implementation are carried out at different depths of the ground. Based on the experience of the participants, the risk of evacuation of passengers, the problem of smoke evacuation, the ease of access of relief forces to the station, the transfer of the injured to the ground, and the process of relief have been more complicated and more difficult at the deeper stations. Moreover, in the occurrence of accidental calamities or emergencies, considering the vulnerability of the station regarding threat of groundwater or being located on a fault will affect the decision to fully evacuate the station. Considering consensus of opinion among the participants, the proximity of the stations to each other, wide area of the platform and available space of the station, the proximity of the station to the ground, and access to fresh air are other features of the station which reduce the vulnerability of the population during emergency evacuation.

P07 "The factors affecting safe evacuation of the passengers in the subway depend on the type of stations and our conditions. On the ground stations, our problems are a bit less, where our station is on the ground, because there is no difference in height, there is fresh air, and it is easier to get access to the relief forces, therefore, in these stations there is less risk for evacuation."P09"... you need to see what time of day the emergency evacuation has occurred, for example, at 11 am maybe there are not a lot of people there; then it may happen at 7:30 am, so the time also matters (in the risk of passenger evacuation)."

According to the safety instructions for evacuation of passengers in the subway, the priority of the location of evacuation is the station platform. In emergencies, the evacuation should be carried out at the nearest safe place, because the electricity of the third rail, the height difference between the wagon floor and the ground, the bumps at the tunnel surface, and low lighting are serious threats to the passengers in tunnel evacuation and challenges the emergency evacuation of the tunnel. Also some of the participants suggested that identifying the access points and safe places for evacuation at each station, determining the safe gathering points on the station map as well as out of the station, and directing the population to these points in emergencies could be effective factors in evacuation of the population with the least damage.

P13 "... Time, appropriate place, and appropriate action matter to us. These are the parameters that must be observed and not be forgotten. If the location of evacuation is not suitable, it may make the situation more critical and that location itself becomes a risk factor."P06" We need to identify the safe places in our area. Like the one you see in the earthquake where you have to find a place to hide, we should also consider the subway incidents that where we can direct the crowd if it happens?"

## Discussion

This study was the first qualitative research to examine the factors affecting the safe evacuation of the population in the subways of Iran from the perspective of all stakeholders including policymakers, executive managers, and the public users of the subway. The most important factors in this study that have changed into serious challenges of emergency evacuation at subway stations were etiquette and manner of interaction and cooperation of passengers, correct and timely decision-making, notification, and the location of emergency evacuation.

The factors of poor culture and interaction of the passengers was a major challenge in this study. Hurrying to enter or exit the train wagons in the form of aggressive behaviors, and lack of respect for the rights of others were proposed as instances for inappropriate behaviors of using subway and this is a challenge addressed in other relevant studies in Iran.^27,28^ Inappropriate interactions among passengers in narrow spaces of wagon and in small area of platforms, especially during peak passenger hours, can lead to one of the potential threats of crowd disaster. As the findings of the present study showed, in emergencies, the inappropriate interaction of passengers with each other and neglect of the orders of the officers resulted in the stampede of passengers towards the exits increasing the risk of damage to the population. The findings of other studies about the effect of the passengers' behavior on the efficiency of emergency evacuation of subway stations also have shown that the spread of panic among passengers could cause secondary incidents, which was consistent with our findings.^8-10,29^ According to the findings of the propagation model of panic in the subway emergency in Shanghai, China, hurrying to exit and escape has led to herding behavior of the populations and stampede, which caused the people to be trampled underfoot especially the disabled ones, dispersing the luggage, and seriously disturbing social order and public transportation.^11^ According to the findings of other studies, one reason for such behavior is that one of the phenomena of collective behavior in emergencies is following a fleeing crowd. In such a situation, the cooperation and interaction of passengers with the station officers as guides who are deployed in emergencies to direct the passengers toward the exits or escape routes increases evacuation efficiency. This cooperation is especially effective for the unfamiliar population with the environment. This predicates the necessity of planning and culture making for further attention and cooperation of the people to the guides on the subway. In agreement with this, the results of the simulation of emergency evacuation of Beijing South Railway Station showed that the guides played a key role in the efficient and orderly evacuation of the passengers.^30^ In another study with the aim of examining the effect of the location and number of guides on population evacuation, the results of the study confirmed that it was essential to use the guide officers in emergency management. Also since more guides does not necessarily lead to better evacuation, in addition to appropriate number of guides in public places, the position of deployment should be correctly set.^31^ In the instructions of emergency evacuation in the context of the present study, the effective interaction of the platform agents with passengers in directing the evacuees towards emergency exit routes and facilitating the evacuation of disabled people or those with special needs were also considered as required tasks for the safe evacuation of subway stations. Also, in several studies in the context of Iran, the inappropriate interaction between people and system custodians in emergencies has been identified as one of the main challenges and obstacles to providing effective responses to traffic crashes victims and providing health services to people in natural disasters.^32-34^ Hence, currently improving the etiquette of using the subway as well as increasing the effective interaction of passengers with platform agents in emergency situations, have been more concerned by the policy makers of crisis prevention and management of Tehran Subway.

Based on the findings of this study, correct and timely decision of authorities for emergency evacuation is one of the organizational challenges and as the main factors affecting the safe evacuation of population. Correct and timely decision of the organization's decision makers to evacuate the station in emergency situations has prevented the transformation of incidents into human disasters in Tehran Subway. For example, in 2012, the incident of ingress of water in Line 4 in Tehran Subway, a huge volume of water flooded the Line 4 in less than a few minutes and the water fully flooded three stations due to the severity of rainfall and the destruction of the wall along the railroad tracks. In this incident, quick and timely decision of the line's custodians to completely evacuate six stations left the incident with no damage or human casualties. In the context of the current study, one of the main reasons that makes the decision for evacuation one of the main challenges of senior and operations managers of the subway, is lack of confidence in the safe evacuation of the population based on the condition-related decisions. The reason is that the decision to announce emergency evacuation, in addition to requiring effective coordination of a wide range of physical and human resources and appropriate infrastructures for safe evacuation, would impose economic, social, and psychological consequences on the subway organization and the evacuees^16^ and, therefore, it is of high importance. In general, the challenge of decision-making for evacuation in large scale has been raised in most public service facilities. In a study on effective factors in decision-making for hospital evacuation, in addition to assessing the threat and consequences of disasters on the hospital infrastructure, the sociopolitical consequences of emergency evacuation were argued,^35^ and suggestions were made for providing decision-making support tools for quick and correct decisions.

The case study of GuoMao Station in Beijing Subway determined the level of warning for hazards and decision for evacuation.^36^ It was suggested that at the time of operation of subway lines, standardized guidelines should be prepared in accordance with the risk assessment and subway specific conditions for each country to clarify the method of decision-making and response to the emergencies.^37,38^ However, so far in subway stations of Tehran, the decisions for emergency response have mainly been made based on the experience of the managers. This implies that designing an assisting tool for quick decision-making for emergency evacuation is required for the line managers and subway station officials, which is the issue that the researchers will explore in their subsequent study of and the results.

The present study indicated that accurate and timely notification in an emergency or incidents was an effective step for responding to emergencies. For accurate notification, mutual understanding between individuals is necessary. The choice of language and use of intelligible words, sound tuning, and the appropriate pause to announce notifications differ under normal circumstances and in an emergency.^39^ In the present study, according to the experience of the participants, the unclearness of warnings and notices, and also misinterpretation of the notifications at the station, were considered as the most important barriers to notification, which has been reported in other studies to lead to safety breach and a threat for the passengers. Several studies have indicated the importance of proper planning for the number and position of the loudspeakers, the acoustic design of the space in terms of sound broadcast and preventing the reflection of sound in crowded public spaces.^39-41^ These factors were examined in a case study of simulated fire in Montreal Highway tunnel, Canada. When the passenger evacuation warning was announced simultaneously from multiple speakers, it was difficult to recognize the words due to time differences between the sounds. Therefore, considering the distance between two adjacent speakers and the sound speed a time-delay system was proposed to solve this problem.^39^

Other factors that cause impaired notification for evacuation of the subway could be inappropriate telecommunication equipment that disturb the timely and effective communication between the subway organization and the support and relief agencies in the event of emergencies. According to the experience of the participants, radio disconnection for the operations teams in deep earth operations and not using a common telecommunications infrastructure for all urban service and relief organizations have been marked as the major causes of disrupting the connection between the organizations in an emergency in Tehran Subway. The problem of telecommunication systems has also been in other areas of disaster management. In a study to identify obstacles and effective facilitators of post-crash management in Iran, inappropriate telecommunication equipment were found to be one of the disrupting factors of coordination and cooperation of the organizations.^33^ This implies that, in order to overcome the challenges of the incidents and normal and emergency evacuation of the population in the subway, it is necessary to use high-performance audio and notification systems for information and safety purposes, and there should be appropriate and constant communication in the subways.

Emergency evacuation in subway stations and underground transportation systems is more challenging as compared with other large public places and ground transportation systems. The construction of subway stations in the deep earth, particular geometry of the tunnel, and confined conditions of the environment revealed the potential risk of evacuation. In an emergency, the location of evacuation is an influential factor in the safety of passengers. In an experimental study of fire and emergency evacuation in the subway tunnel, lighting conditions, ground surface material, tunnel roughness and bumps, and smoke were listed as the factors affecting the movement speed of the evacuees. On the other hand, in a tunnel the distance to the safe place, i.e., tunnel entrances, and emergency exits or safe havens is usually very long.42 In the context of the present study, regarding the failure to execute a number of stations which were designed but not implemented in the initial map and general structure of Tehran Subway, the distance between some stations is greater than the safety standards. Long distances between the stations might further complicate the rescue operation in the tunnels and affect the risk of no delivery of rescue services for evacuation as well as the risk of harm to the population at risk. Moreover, due to height difference between the wagon floor and the ground in the tunnel, there are serious limitations for detrainment to the ground, which can simply create bottlenecks in evacuation conditions. In a study conducted on China Subway to identify the effect of the train stopping places on the executive performance of passenger evacuation, the process of evacuation of passengers was simulated in nine different locations of the train stop in a subway tunnel. The simulation results were evaluated by several performance indicators related to the evacuation time and showed that various scenarios of train breakdown and detrainment led to diverse evacuation processes and affect the efficiency and safety of evacuation.^43^ Consequently, depending on where the emergency evacuation is located on the subway route, protecting passengers' safety is a major concern for design engineers, subway operating companies, and the governments.

**Strengths and limitations of the study**

Contrary to quantitative studies, a limitation of the present study could be small number of participants, however, the participants in this study provided us with rich data from a wide range of stakeholders, which is an important advantage and utility of qualitative study from a fresh perspective of the phenomena of interest.^44^ Since the current research is based on stakeholders experience, it has the potential to present the real challenges of the organization and the role of people in emergency evacuation. Also this is one of the few studies with qualitative approach to highlight the ways of improving the current status.

## Conclusion

The participants in this study believed that improving the culture, effective interaction and cooperation of the passengers in an emergency evacuation was an affecting factor in safe evacuation of the stations. However, in construction and operation of new stations most efforts should be concentrated on establishing emergency evacuation infrastructures such as escape and emergency exit routes at the stations and the tunnels in accordance with the population growth and real needs of the passengers. In recent years, the policy makers' approach toward operation of subway lines has been focused on expanding the subway transit network in the city in order to improve the accessibility of subway for the passengers and serving the largest number of passengers. However, regarding population growth and inappropriateness of subway evacuation resources and infrastructures, the attitude of the policy makers is supposed to be upgraded from quantitatively improving the transportation services to enhancing the quality of trip and passenger safety. Utilizing emergency telecommunication equipment in emergencies with the feature of producing integrated frequency among subway operations teams and urban relief teams in order to establish constant communications and effective relief services, and also timely notification of station evacuation at the safe time were recommended in this study.

The results of this study indicated that in emergency evacuation with the approach of passenger safety, organization preparedness and training of people for response to emergency and evacuation should be more concentrated. In addition, designing emergency decision-making tools for emergency evacuation was suggested to improve correct and quick decision-making skills of the station officials, which is currently under way.

**Acknowledgements**

The School of Public Health and Safety, Shahid Beheshti University of Medical Sciences supported this study both financially and administratively. The authors acknowledge the contributions of Tehran Urban and Suburban Railway Operations Company members and operations managers and personnel of subway stations. The authors would like to thank Hossein Amirahmadi and Mohammad Sadegh Mohammadi for their contributions. We also express our gratitude to Crisis Prevention and Management Organization of Tehran Subway and station police officers in Tehran Urban and Suburban Railway Organization.

## References

[1] Zeng S, Zha XX, Chen YY, Jiang RJ (2014). Safe Evacuation from Subway Station under Platform Train Fire. Applied Mechanics and Materials.

[2] Zhong M, Shi C, Tu X, Fu T, He L (2008). Study of the human evacuation simulation of metro fire safety analysis in China. Journal of Loss Prevention in the Process Industries.

[3] Shi CL, Zhong MH, Nong XZ, He L, Shi JH, Feng GG, Saf Sci (2012). Modeling and safety strategy of passenger evacuation in a metro station in China. Saf Sci.

[4] Ceng S. Studies on intelligent evacuation modeland simulation of fire in urban underground building. Master Thesis. South China University of Technology, People's Republic of China, Ann Arbor, China, 2009.

[5] Lovreglio R. Modelling Decision-Making in Fire Evacuation based on Random Utility Theory. Interpolitechnical School of Doctorate. PhD Thesis. Politechnic of Bari, Italy, 2016:192.

[6] Zhang L, Wu X, Liu M, Liu W, Ashuri B (2019). Discovering worst fire scenarios in subway stations: A simulation approach. Autom Constr.

[7] Nie HJ. Study on safety egress in subway fire. Master Thesis. Northeastern University, People's Republic of China, Ann Arbor, 2008.

[8] Wang CX, Suo X, Lyu SR, Yang K (2015). Research on panic degree model of emergency evacuation from subway. Zhongguo Anquan Kexue Xuebao China Saf Sci J.

[9] Wang Jh, Yan W-y, Zhi Y-r, Jiang J-c (2016). Investigation of the panic psychology and behaviors of evacuation crowds in subway emergencies. Procedia Engineering.

[10] Wang J, Chen M, Yan W, Zhi Y, Wang Z (2017). A utility threshold model of herding–panic behavior in evacuation under emergencies based on complex network theory. Simulation.

[11] Zhao H, Jiang J, Xu R, Ye Y. SIRS Model of Passengers' Panic Propagation under Self-Organization Circumstance in the Subway Emergency. Mathematical Problems in Engineering. 2014;2014.

[12] System afcA. automated fare collection (AFC) system data. In: Co. TUSRO, ed. Tehran, Iran, 2017.

[13] Ma L, Chen B, Qiu S, Li Z, Qiu X (2017). Agent-based modeling of emergency evacuation in a railway station square under sarin terrorist attack. International Journal of Modeling, Simulation, and Scientific Computing.

[14] Li Z-y, Tang M, Liang D, Zhao Z (2016). Numerical simulation of evacuation in a subway station. Procedia Engineering.

[15] Zhang LM, Liu MJ, Wu XG, AbouRizk SM (2016). Simulation-based route planning for pedestrian evacuation in metro stations: A case study. Autom Constr.

[16] Wang J-h, Sun J-h (2014). Principal aspects regarding to the emergency evacuation of large-scale crowds: a brief review of literatures until 2010. Procedia Engineering.

[17] Xiaojun Z, Xueying Y (2013). Study on safety evacuation time for passengers in subway station and its application. Advanced Materials Research.

[18] Jackiva IY, Savrasovs M, Gromule V, Zemljanikins V (2016). Passenger Terminal Safety: Simulation Modelling as Decision Support Tool. Procedia Engineering.

[19] Li D, Han B (2006). Modeling and simulation pedestrian evacuation process in mass transit railway stations. International Symposium on Safety Science and Technology.

[20] Li Y. Research on evacuation management of urban rail transportation center based on path assignment. Master Thesis. Beijing Jiaotong University People's Republic of China, Ann Arbor, China, 2010.

[21] Elo S, Kääriäinen M, Kanste O, Pölkki T, Utriainen K, Kyngäs H (2014). Qualitative content analysis: A focus on trustworthiness. Sage Open.

[22] Saunders B, Sim J, Kingstone T, Baker S, Waterfield J, Bartlam B (2018). Saturation in qualitative research: exploring its conceptualization and operationalization. Quality & Quantity.

[23] Nieswiadomy RM, Bailey C. Foundations of Nursing Research. Pearson; 2017.

[24] Bengtsson M (2016). How to plan and perform a qualitative study using content analysis. NursingPlus Open.

[25] Graneheim UH, Lundman B (2004). Qualitative content analysis in nursing research: concepts, procedures and measures to achieve trustworthiness. Nurse Eeducation Today.

[26] Sciences ECoSBUoM. official letter. IR.SBMU.RETECH.REC.1396.201. Shahid Beheshti University of Medical Sciences,Tehran, Iran; 15 Septamber 2017.

[27] Lankarani KB, Sarikhani Y, Heydari ST, Joulaie H, Maharlouei N, Peimani P (2014). Traffic accidents in Iran, a decade of progress but still challenges ahead. Medical Journal of the Islamic Republic of Iran.

[28] Khorasani-Zavareh D, Mohammadi R, Khankeh HR, Laflamme L, Bikmoradi A, Haglund BJ (2009). The requirements and challenges in preventing of road traffic injury in Iran. A qualitative study. BMC Public Health.

[29] Li F, Chen S, Wang X, Feng F (2014). Pedestrian evacuation modeling and simulation on metro platforms considering panic impacts. Procedia-Social and Behavioral Sciences.

[30] Yang X, Dong H, Wang Q, Chen Y, Hu X (2014). Guided crowd dynamics via modified social force model. Physica A: Statistical Mechanics and its Applications.

[31] Yang X, Dong H, Yao X, Sun X, Wang Q, Zhou M (2015). Necessity of guides in pedestrian emergency evacuation. Physica A: Statistical Mechanics and its Applications.

[32] Haghparast-Bidgoli H, Hasselberg M, Khankeh H, Khorasani-Zavareh D, Johansson E (2010). Barriers and facilitators to provide effective pre-hospital trauma care for road traffic injury victims in Iran: a grounded theory approach. BMC Emergency Medicine.

[33] Khorasani-Zavareh D, Khankeh HR, Mohammadi R, Laflamme L, Bikmoradi A, Haglund BJ (2009). Post-crash management of road traffic injury victims in Iran. Stakeholders' views on current barriers and potential facilitators. BMC Emergency Medicine.

[34] Khankeh HR, Khorasani-Zavareh D, Johanson E, Mohammadi R, Ahmadi F, Mohammadi R (2011). Disaster health-related challenges and requirements: a grounded theory study in Iran. Prehospital and Disaster Medicine.

[35] Yaghoubi T, Ardalan A, Zavareh DK, Khankeh H, Nejati A, Ebadi A (2017). Decision-making on hospital emergency evacuation in disasters and emergen-cies: findings from a systematic review. Iranian Red Crescent Medical Journal.

[36] Lu K, Han B (2016). Congestion risk evaluation and precaution of passenger flow in metro stations. Open Civil Engineering Journal.

[B37] Association NFP. NFPA 130: Standard for Fixed Guideway Transit and Passenger Rail Systems. NFPA; 2003.

[38] Chen T, Zhang SY, Zhao LZ, Xia JJ, Fu XC, Bao ZM, et al. Comparison of safety equipment between London underground and Beijing subway. IOP Conference Series: Earth and Environmental Science; 2017:1-9.

[39] Tachibana H. Public space acoustics for information and safety. Proceedings of Meetings on Acoustics; 2013.

[40] Chen Y-z, Yang R, Liu Y (2014). Strategy study on mass evacuation with LBS information. International Conference on Web-Age Information Management. Springer.

[41] Zhao H, Winter S, Tomko M (2017). Integrating decentralized indoor evacuation with information depositories in the field. ISPRS International Journal of Geo-Information.

[42] Fridolf K, Nilsson D, Frantzich H (2013). Fire Evacuation in Underground Transportation Systems: A Review of Accidents and Empirical Research. Fire Technology.

[43] Wang WL, Jacqueline Lo TY (2014). A simulation study on passenger escape in rail tunnels. Procedia Engineering.

[44] Khankeh H, Ranjbar M, Khorasani-Zavareh D, Zargham-Boroujeni A, Johansson E (2015). Challenges in conducting qualitative research in health: A conceptual paper. Iranian Journal of Nursing And Midwifery Research.

